# Homeostatic control of brain function – new approaches to understand epileptogenesis

**DOI:** 10.3389/fncel.2013.00109

**Published:** 2013-07-16

**Authors:** Detlev Boison, Ursula S. Sandau, David N. Ruskin, Masahito Kawamura, Susan A. Masino

**Affiliations:** ^1^Robert Stone Dow Neurobiology Laboratories, Legacy Research InstitutePortland, OR, USA; ^2^Department of Psychology and Neuroscience Program, Trinity CollegeHartford, CT, USA; ^3^Department of Pharmacology, Jikei University School of MedicineMinato-ku, Tokyo, Japan

**Keywords:** adenosine, glial cells, ketogenic diet, mitochondrial bioenergetics and physiology, DNA methylation, transmethylation pathway, epileptogenesis, homeostasis

## Abstract

Neuronal excitability of the brain and ongoing homeostasis depend not only on intrinsic neuronal properties, but also on external environmental factors; together these determine the functionality of neuronal networks. Homeostatic factors become critically important during epileptogenesis, a process that involves complex disruption of self-regulatory mechanisms. Here we focus on the bioenergetic homeostatic network regulator adenosine, a purine nucleoside whose availability is largely regulated by astrocytes. Endogenous adenosine modulates complex network function through multiple mechanisms including adenosine receptor-mediated pathways, mitochondrial bioenergetics, and adenosine receptor-independent changes to the epigenome. Accumulating evidence from our laboratories shows that disruption of adenosine homeostasis plays a major role in epileptogenesis. Conversely, we have found that reconstruction of adenosine’s homeostatic functions provides new hope for the prevention of epileptogenesis. We will discuss how adenosine-based therapeutic approaches may interfere with epileptogenesis on an epigenetic level, and how dietary interventions can be used to restore network homeostasis in the brain. We conclude that reconstruction of homeostatic functions in the brain offers a new conceptual advance for the treatment of neurological conditions which goes far beyond current target-centric treatment approaches.

## INTRODUCTION

Epileptogenesis is a complex process that not only involves changes in neuronal excitability and circuitry, but also changes in glial physiology and in the homeostatic environment in which neurons need to survive and to function properly (Kunz, 2002; Borges et al., 2003; David et al., 2009; Ravizza et al., 2011; Devinsky et al., 2013). Characterized by abnormal and excessive neuronal firing, each seizure represents a rapid loss of homeostatic equilibrium, with altered energy and molecular gradients, and a corresponding interruption of normal behavior and consciousness. Because having a seizure can increase the likelihood of future seizures, seizures themselves contribute to epileptogenesis. Similarly, conditions that can precipitate epilepsy – such as traumatic brain injury, and diseases in which epilepsy can be comorbid – such as Alzheimer’s disease, are accompanied by a chronic loss of homeostatic function. Therefore, the loss of homeostasis associated with epilepsy is found acutely during the seizure or precipitating event, and also during the chronic process of epileptogenesis.

Unfortunately, the pursuit of neurocentric therapeutic targets did not yield any antiepileptogenic therapies to date (Loscher and Brandt, 2010). In contrast, a revised understanding of epilepsy as a complex syndrome of disrupted network homeostasis may yield novel therapeutic avenues to halt, disrupt, or even reverse the process of epileptogenesis. Key elements to consider with the goal of restoring network homeostasis are glial function and metabolism. Akin to seizures themselves, which have negative acute and chronic effects, restoring homeostasis can benefit acute brain function and avert the progressive process of epileptogenesis.

Glial cells play a major role in the homeostatic state of the brain by regulating the ambient concentration of synaptic neurotransmitters; modulating the permeability of the blood brain barrier (BBB) through astrocyte–endothelial interactions; regulating cerebral blood flow; and microglial control of brain immunity. Thereby, glial cells directly influence brain function on multiple levels including neuronal excitability and synaptic transmission; delivering energy substrates from the periphery; and recovery from injury or infection (Eulenburg and Gomeza, 2010; Kofler and Wiley, 2011; Petzold and Murthy, 2011; Santello et al., 2012). As a consequence, disruptions to normal glial cell function as observed in neurological disorders with a gliotic pathology has widespread deleterious ramifications that contribute to disease progression and maintenance through changes in synaptic activity, BBB permeability, brain immunity, and inflammation (Carmignoto and Haydon, 2012; Coulter and Eid, 2012; Kovacs et al., 2012).

Within human epileptic foci the most prominent pathological finding is gliosis, with reports of reactive astrocytes, microglia, glial scars, and/or gliomas being present (Kallioinen et al., 1987; Kurzwelly et al., 2010; Butler et al., 2013). Pathological glial cells have been associated with a spectrum of different neurological diseases that result in epilepsy including mesial temporal lobe epilepsy with hippocampal sclerosis (mTLE), focal cortical dysplasia (FCD), tuberous sclerosis complex (TSC), and Rasmussen’s encephalitis (Sosunov et al., 2008; Malmgren and Thom, 2012; Butler et al., 2013). Concurrent with a gliotic pathology, studies from both epileptic patients and rodent models for epilepsy have identified abnormal glial cell activity as a contributing factor to seizures and/or epileptogenesis.

Alongside gliosis, it is increasingly appreciated that epilepsy is a global dysregulation involving metabolic dysfunction (DiMauro et al., 2002; Kunz, 2002), and, furthermore, that metabolic dysfunction is common in neurological disorders including neurodegenerative (Sas et al., 2007) and psychiatric disorders (Rezin et al., 2009). The ketogenic diet, developed as a treatment for epilepsy nearly 100 years ago, is a highly successful metabolic strategy now moving broadly into translational work for a variety of neurological disorders. Multiple lines of evidence suggest that key mechanisms underlying the acute anticonvulsant effects of a ketogenic diet may be adenosine acting via K_ATP_ channels. These findings highlight the potential for altered metabolism restoring and maintaining homeostasis in the central nervous system (CNS), and have implications for exploring the prevention and treatment of neurological disorders using strategies other that traditional neurocentric approaches.

Here we outline homeostatic therapeutic strategies with a focus on adenosine, an endogenous bioenergetic homeostatic regulator; multiple lines of evidence suggest that adenosine homeostasis is a key factor in preventing and stopping seizures. This review will first discuss glial mechanisms of epileptogenesis with emphasis on disruptions of adenosine homeostasis. Based on these mechanisms, we identify glia-centric therapeutic strategies that treat epilepsy through restoring normal brain homeostasis. We then highlight recent evidence regarding the role of adenosine in the process of epileptogenesis and describe metabolic therapy via a ketogenic diet, which may restore adenosine homeostasis.

## GLIAL MECHANISMS IN EPILEPTOGENESIS

**FIGURE 1:**
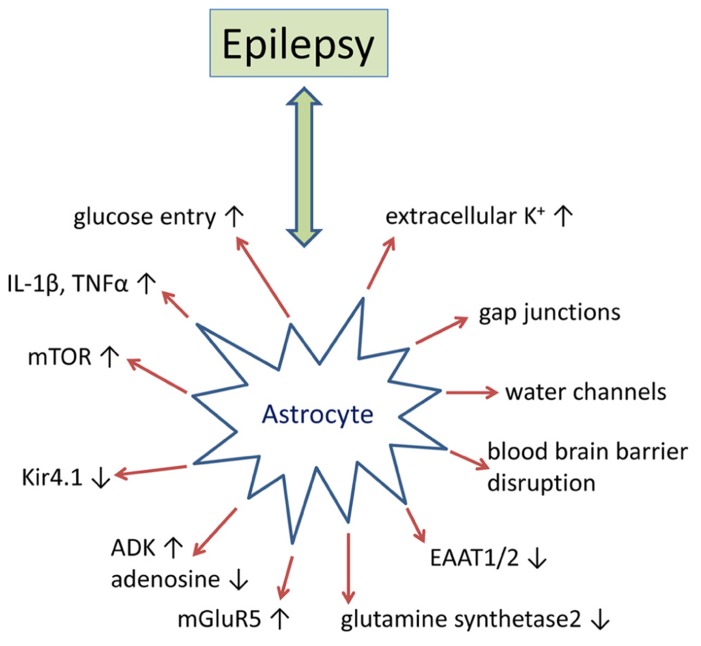
** Epilepsy-associated alteration of astrocyte-based homeostatic functions.** Summary of key pathophysiological alterations of astrocytes as found in animal models of epilepsy and/or in human epilepsy. For details, please refer to main text.

Historically epilepsy research has predominantly focused on disruptions to normal neuronal function as the primary etiology. However, there is a substantial amount of evidence that implicate glial dysfunction as a major contributing factor to epileptogenesis (**Figure 1**). Astrocytes regulate or modulate a number of neuronal functions including excitability, synaptic transmission, and plasticity. As a consequence, the presence of reactive astrocytes, as found in patients with mTLE, FCD, and TSC, disrupts normal neuronal activity that either promotes epileptogenesis or decreases seizure threshold (Sosunov et al., 2008; Miyata et al., 2013). Multiple mechanisms by which reactive astrocytes may directly modulate neuronal activity at the synaptic cleft have been proposed. These include, but are not limited to (i) increases in neuronal excitability caused by decreased adenosine tone; increased synaptic glutamate levels; changes in the extracellular space (ECS) volume and K^+^ ion concentration; and (ii) modulation of synaptic transmission through glutamate, adenosine triphosphate (ATP), adenosine, gamma-aminobutyric acid (GABA), or D-serine from astrocytes (Devinsky et al., 2013). While multiple glial mechanisms of epileptogenesis have been investigated, disruption of adenosine homeostasis has consistently been identified as sufficient for seizure generation and has proven to be an effective therapeutic target for seizure suppression and stopping disease progression (Boison, 2013). Here we will further review the supporting data from human epilepsy and rodent models of epilepsy that pertain to astroglial-mediated disruptions in synaptic transmission as a mechanism for epileptogenesis, with a more thorough discussion of adenosine homeostasis in epilepsy to follow.

### GLUTAMATE HOMEOSTASIS

Reactive astrocytes cause neuronal hyperexcitability through increased synaptic glutamate and K^+^ levels and decreased ECS volume. Decreased astrocyte-mediated glutamate uptake and glutamate to glutamine conversion have been proposed to increase synaptic glutamate in the gliotic hippocampus (Cavus et al., 2005). Increased levels of synaptic glutamate may in part be attributed to reduced expression of glutamate transporters within reactive astrocytes. High affinity glutamate transporters (2–90 μM) are concentrated on the astrocyte membrane and are integral to maintaining a low glutamate tone within the synaptic cleft. Thus, a decrease in astrocyte glutamate transporters may increase neuronal hyperexcitability and decrease seizure threshold. A patient diagnosed with spontaneous seizures was found to have a mutation in the human gene SLC1A3, resulting in decreased EAAT-1 protein expression and reduced capacity for glutamate uptake (Jen et al., 2005). A substantial decrease in both astrocyte glutamate transporters, EAAT-1 and EAAT-2, has also been identified in resected mTLE hippocampi (Sarac et al., 2009). However, this finding has not been reproduced in other studies (Tessler et al., 1999; Eid et al., 2004). Research with transgenic mice further implicates deregulation of astrocyte-mediated glutamate uptake as a contributing factor to epilepsy. A mouse model of TSC with progressive epilepsy was found to have decreased glutamate/aspartate transporter (GLAST) and glial glutamate transporter 1 (GLT-1) protein levels (genetic equivalents to EEAT-1 an EAAT-2, respectively) and glutamate transporter currents (Wong et al., 2003). In addition, GLT-1 and GLAST knockout mice have a decreased pentylenetetrazol (PTZ) seizure threshold. The GLT-1 knockouts also exhibit spontaneous lethal seizures and have increased levels of synaptic glutamate (Tanaka et al., 1997; Watanabe et al., 1999). Downregulation of glutamine synthetase (GS) within reactive astrocytes has also been postulated as a potential cause for the increased synaptic glutamate tone observed in the epileptic hippocampus (Eid et al., 2004). GS primarily resides in the astrocyte cytoplasm and is responsible for the ATP-dependent conversion of glutamate to glutamine. GS protein and enzymatic activity are profoundly decreased, 40 and 38%, respectively, in the hippocampus of mTLE patients with the greatest reduction observed in proliferating astrocytes (Eid et al., 2004). Causative evidence that reduced GS activity is sufficient for epileptogenesis is from a pharmacological study with rats that developed seizures and neuropathology reminiscent of mTLE when chronically infused with the GS in inhibitor methionine sulfoximine (Wang et al., 2009). Furthermore, a mutation in the gene encoding GS has been linked to child with epilepsy (Haberle et al., 2011).

### WATER AND POTASSIUM HOMEOSTASIS

The state of neuron excitability is also tightly coupled to the ECS volume and associated K^+^ homeostasis (Schwartzkroin et al., 1998). More specifically, hypoosmolarity treatment reduces the ECS volume and increases neuron excitability and epileptiform activity; while hyperosmolarity treatment has the reverse effect. Aquaporin 4 (AQP4) is a water transport channel that is expressed within glial cells and which is integral to regulating ECS volume and implicated in epileptogenesis (Binder et al., 2012). AQP4 is normally localized to both the perivascular endfeet and within perisynaptic processes of astrocytes where it permits the bidirectional flow of water from the ECS to the blood (Nielsen et al., 1997; Rash et al., 1998; Nagelhus et al., 2004). In human mTLE brain specimens, AQP4 is redistributed primarily to the perisynaptic processes, which has been hypothesized to be a contributing factor of hyperexcitability through dysregulating water and K^+^ homeostasis (Eid et al., 2005). Research with transgenic AQP4 knockout mice support this hypothesis as they have increased ECS volume and are less susceptible to PTZ-induced seizures (Binder et al., 2004a,b). Glial-mediated water flow is also tightly coupled to K^+^ transport from the ECS through the inward rectifying K^+^ channel, Kir4.1, that is colocalized with AQP4 on the astrocyte membrane (Hsu et al., 2011). Similar to ECS volume, changes in the K^+^ concentration influence neuronal excitability with millimolar increases in ECS K^+^ exacerbating epileptiform activity (Feng and Durand, 2006). Human polymorphisms in KCNJ10, the gene that encodes Kir4.1, are associated with epilepsy and a glial specific deletion of Kir4.1 in mice reduces K^+^ clearance from the synaptic cleft (Heinemann et al., 2000; Haj-Yasein et al., 2011). Dysregulation of AQP4 might also be linked to cholinergic imbalances in epilepsy, since overexpression of synaptic acetylcholinesterase has been associated with overexpression of AQP4 (Meshorer et al., 2005)

### GLIOTRANSMITTER HOMEOSTASIS

Aside from increasing neuronal excitability, astrogliosis disrupts synapse homeostasis through dysregulation of transmitter release from astrocytes. The list of transmitters proposed to be released by astrocytes includes glutamate, D-serine, ATP, adenosine, and GABA (Devinsky et al., 2013). In regards to adenosine homeostasis, astrocytes express two types of equilibrative nucleoside transporters, which mediate transport based on the concentration gradient of adenosine (Baldwin et al., 2004; Gray et al., 2004; Guillen-Gomez et al., 2004; Peng et al., 2005; Alanko et al., 2006). Adenosine in synapses of CA1 pyramidal neurons can be generated in response to high frequency stimulation that induces a Ca^2^^+^-mediated release of ATP from astrocytes through either vesicular transport or hemichannels (Cotrina et al., 1998; Zhang et al., 2003; Pascual et al., 2005; Kang et al., 2008) or the direct release of adenosine from neurons (Lovatt et al., 2012). Once in the synaptic cleft ATP is rapidly converted to adenosine by a series of ectonucleotidases (Zimmermann, 2000). Ca^2^^+^ waves within astrocytes have also been linked to glutamate and D-serine release. Within the epileptic brain Ca^2^^+^ signaling may regulate the glutamate-induced paroxysmal deporalization shift, which is the intracellular analog to the interictal spike (Tian et al., 2005). However, results from a separate study suggest that astrocytes may initiate seizures and not contribute to interictal activity (Gomez-Gonzalo et al., 2010).

### GROWTH FACTORS

Status epilepticus (SE) can induce a wide range of growth factors, neurotrophins, and transcription factors (Grabenstatter et al., 2012). Increased brain-derived neurotrophic factor (BDNF) in particular has been linked to epileptogenesis (Grabenstatter et al., 2012). In neurons, BDNF was shown to activate the Janus kinase/signal transducer and activator of transcription (JAK/STAT) pathway, cyclic adenosine monophosphate (cAMP) response element binding protein (CREB), inducible cAMP early repressor (ICER), and early growth response factors (Egrf) that induce a shift in the expression of specific subunits of the GABA_A_R as well as the expression levels of *N*-methyl-D-aspartate receptors (NMDARs; Roberts et al., 2006; Lund et al., 2008; Kim et al., 2012). In astrocytes, activation of the trkB receptors by BDNF has been linked to the development of astrogliosis, a mechanism that is also influenced by transactivation of the TrkB receptor through the adenosine A_2A_R (Brambilla et al., 2003). Whereas the role of growth factors on neuronal function is well documented, growth factor-dependent mechanisms that contribute to epileptogenesis via the disruption of glial homeostatic functions are not well established.

### BLOOD BRAIN BARRIER

The breakdown of the BBB leading to albumin extravasation has directly been linked to epileptogenesis (Heinemann et al., 2012). Albumin is a potent astroglial activator through stimulation of transforming growth factor beta (TGF-β) signaling and activation of the SMAD-2/5 pathway (Ivens et al., 2007; Cacheaux et al., 2009). BBB disruption also triggered expression changes of genes associated with the TGF-β pathway, early astrocyte activation, inflammation, and reduced buffering for glutamate and K^+^ (Cacheaux et al., 2009; David et al., 2009). The reduced buffering capacity of transformed astrocytes for glutamate and K^+^ appears to be most critical during repetitive activation. Experimental blockade of TGF-β signaling following BBB disruption decreased those transcriptional responses and prevented epileptogenesis. BBB disruption has been demonstrated in patients with posttraumatic epilepsy (Tomkins et al., 2008) and in patients with brain tumors who developed epilepsy (Marchi et al., 2007). Thus, pathogenetic neurovascular interactions which involve astroglial dysfunction, changes in the immune response, and gene expression changes that promote neuronal hyperexcitability may play a critical role in epileptogenesis. Consequently, BBB disruption might constitute a valuable biomarker for the prediction of epileptogenesis following an insult to the brain.

### IMMUNOLOGICAL RESPONSES

Inflammatory processes play important roles in the pathogenesis of epilepsy (Aronica et al., 2012). Molecules linked to inflammatory reactions, such as TNF-α or prostaglandins, control the release of glutamate from astrocytes (Rossi and Volterra, 2009). The activation of pro-inflammatory pathways, such as the interleukin-1/Toll-like receptor (IL-1R/TLR) pathway, appear to be involved in the precipitation and recurrence of seizures in rodent models of epilepsy (Vezzani et al., 2011a). Importantly, components of these pathways were found to be overexpressed in surgically resected specimens from human TLE (Ravizza et al., 2008). Activation of the IL-1R/TLR pathway can increase excitability of the brain by the induction of post-translational changes in voltage- and ligand-gated ion channels. Among the endogenous ligands are danger signals, such as high mobility group box 1 (HMGB1), which can be released from injured or activated cells (Vezzani et al., 2011b). HMGB1 is the endogenous ligand of TLR4 and normally bound to chromatin. However, HMGB1 can be released into the ECS following either cell damage or neuronal hyperexcitability. The pro-epileptogenic role of HMGB1 is supported by recent data showing that blockade of the TLR4 pathway significantly delays seizure onset (Maroso et al., 2010). Likewise, engineered mice with defects in the IL-1R/TLR signaling pathway are intrinsically resistant to seizures (Vezzani et al., 2011b). Intriguingly, activation of the IL-1R/TLR pathway may alter the permeability properties of the BBB via the production of cytokines and prostaglandins, promoting brain extravasation of albumin (Cacheaux et al., 2009). Thus, inflammatory processes and disruption of the BBB might form a self-perpetuating vicious cycle supporting chronic hyperexcitability of the brain via compromised astrocyte function on multiple levels.

## ADENOSINE – A HOMEOSTATIC NETWORK REGULATOR

The purine ribonucleoside adenosine has early evolutionary origins and likely played already a role in prebiotic evolution (Oro and Kimball, 1961). Importantly, adenosine is not only part of the energy metabolite ATP but also of RNA, the nucleic acid thought to be at the origin of life (Lahav, 1993; Dworkin et al., 2003; Robertson and Joyce, 2012). While ATP reflects the energy pool in the environment, RNA reflects the metabolic activities of a cell. Thus, adenosine assumes a central place between energy availability and metabolic demands and has therefore been termed a retaliatory metabolite (Newby et al., 1985). It is fair to assume that adenosine played an early evolutionary role as key bioenergetic network regulator central to the energy homeostasis of a cell. The early evolutionary principle to conserve energy was likely a rise in adenosine as a consequence to ATP depletion and to use the increase in adenosine as a negative feedback regulator to attenuate all cellular activities that consume energy. This early evolutionary principle is omnipresent in all living systems and in every human organ. In the brain, epileptic seizures cause a rapid drop in energy, which results in the generation of adenosine levels that can exceed the baseline level more than 40 times (During and Spencer, 1992); it is this rise in adenosine that acts as endogenous terminator of seizures and which is responsible for the postictal refractoriness that normally follows a seizure (Lado and Moshe, 2008). Consequently, adenosine augmentation therapies constitute a promising avenue for seizure control (Boison, 2007). Seizure suppression by adenosine depends on the activation of G-protein coupled adenosine A_1_ receptors (Fredholm et al., 2005); however, new evidence suggests that adenosine retains important adenosine receptor-independent regulatory functions, which are based on interactions with mitochondrial bioenergetics, interference with biochemical enzyme reactions, and epigenetic functions. Thereby adenosine assumes a unique role as homeostatic network regulator.

### ADENOSINE RECEPTOR-DEPENDENT PATHWAYS

A number of adenosine’s actions are mediated by a group of specific receptors, G protein-linked transmembrane proteins of the P1 family, distinguished from the P2 ATP receptor family. Four members of the P1 class have been cloned in mammals: A_1_R, A_2a_R, A_2b_R, and A_3_R (Fredholm et al., 2011), and not surprisingly, given the ancient biological origin of adenosine, homologous genes have been found in numerous other animal groups (Sazanov et al., 2000; Petersen et al., 2003; Dolezelova et al., 2007; Boehmler et al., 2009; Malik and Buck, 2010). These receptors have biochemical specificity as each acts through a particular set of G proteins to influence second messengers: for the classical second messenger cAMP, A_1_R and A_3_R activation inhibits its production, whereas A_2a_R and A_2b_R activation are stimulatory (Fredholm et al., 2011). Other second messengers such as diacylglycerol, inositol triphosphate, and Ca^2^^+^ are also modulated. Each receptor presents a distinct pharmacology, and each has a particular distribution in tissues and cell types. For instance, A_1_Rs are expressed most highly in brain, whereas A_2b_Rs and A_3_Rs have their highest expression in the periphery (Dixon et al., 1996). Within the brain, A_1_Rs are widespread with particularly high levels in the limbic system, whereas A_2a_Rs are expressed mostly in the basal ganglia (Dixon et al., 1996).

Adenosine can have powerful receptor-mediated effects on synaptic transmission in the brain (Fredholm et al., 2011). Presynaptic A_1_Rs inhibit synaptic release of most, if not all, neurotransmitters, with an apparently greater effect on excitatory transmission. Thus, if adenosine levels are raised sufficiently, synaptic transmission can be blocked altogether. On the postsynaptic side, A_1_Rs hyperpolarize membranes by opening inwardly rectifying K^+^ channels. These combined A_1_R effects strongly dampen the synaptic network, and undoubtedly play a major role in the efficacious anticonvulsant effect of adenosine and A_1_R agonists (Boison, 2007). The effect of A_2a_Rs on network excitability is less clear, and more anatomically restricted, but if seizures reflect brain network imbalance, then one seizure model suggests that A_1_Rs and A_2a_Rs may cooperate to promote homeostasis (De Sarro et al., 1999).

### MITOCHONDRIAL BIOENERGETICS

Mitochondria generate ATP via oxidative phosphorylation, and this is the main pathway for generating this critical cell energy molecule. Regarding the relationship between adenosine and ATP, intracellular adenosine is dephosphorylated from AMP by cytosolic 5′-nucleotidase and is converted back to AMP via adenosine kinase (ADK). The adenosine-AMP cycle is linked to ADP and ATP with adenylate kinase. Thus, adenosine is linked tightly to energy metabolism. Whereas mitochondrial uncouplers decrease ATP and increase adenosine (via net dephosphorylation of ATP), mitochondrial enhancers, or other strategies, which enhance ATP also appear to increase adenosine. Therefore, improving mitochondrial bioenergetics has the potential to offer dual benefits of improving metabolic dysfunction and restoring adenosine homeostasis.

Adenosine triphosphate is released from various pathways including vesicular release (Coco et al., 2003; Pascual et al., 2005), gap junction hemichannels (Kang et al., 2008) and chloride channels (Anderson et al., 2004), and hydrolyzed to adenosine by a series of ectonucleotidases (Zimmermann, 2000). Interestingly astrocytes express all types of ATP-releasing proteins and are capable of releasing ATP from these pathways simultaneously (Garre et al., 2010). After ATP is dephosphorylated, extracellular adenosine is salvaged into the intracellular space by equilibrative nucleoside transporters and/or concentrative nucleoside transporters (Latini and Pedata, 2001). However, it has also been reported that these nucleoside transporters release adenosine with various types of metabolic stress (Lloyd et al., 1993; Frenguelli et al., 2007). Adenosine-AMP cycles and bidirectional adenosine uptake and release via nucleoside transporters maintain adenosine homeostasis. Therefore, changes in extracellular adenosine due to adenosine and/or ATP release can alter adenosine receptor signaling described above, and experimentally increasing intracellular adenosine or ATP concentration can increase the activity of adenosine receptors (Brundege and Dunwiddie, 1996; Kawamura et al., 2010).

Taken together, adenosine’s role in maintaining homeostasis interacts directly with mitochondrial bioenergetics and energy metabolism (Newby, 1984; Newby et al., 1985; Sommerschild and Kirkeboen, 2000). The intracellular concentration of ATP is nearly 50 times higher than that of AMP (Arch and Newsholme, 1978) and about 10,000 times higher than that of adenosine (Pazzagli et al., 1995; Delaney and Geiger, 1996). Thus, minor decreases in intracellular ATP leads to a large rise of intracellular adenosine level. Thus, various excitatory stimuli cause decreased brain energy and a subsequent increase in adenosine (Shepel et al., 2005). It has been reported that increases in intramitochondrial AMP cause adenosine production in the purified mitochondria, and thus extramitochondrial adenosine levels increase in a time-dependent manner, suggesting a concentration-dependent adenosine output from mitochondria by diffusion or facilitated diffusion (Raatikainen et al., 1992). Conversely, the cytosolic adenosine formation with a balance of cytoplasmic ADK and cytosolic 5′-nucleotidase might influence mitochondrial adenosine production and affect mitochondrial bioenergetics. This interpretation is supported by a severe mitochondrial pathology in ADK knockout mice (Boison et al., 2002). As a “retaliatory metabolite” adenosine is thought to be one of the key links between neuronal network homeostasis and mitochondrial bioenergetics with both adenosine receptor-dependent and -independent pathways.

### EPIGENETICS

Modifications to the epigenome that include changes in DNA methylation, histone tail modifications, and incorporation of histone variants are mechanisms by which network homeostasis can be dramatically altered and consequently change the entire gene expression profile of a tissue. There are a number of epilepsy-associated neurological diseases that are directly attributed to primary genetic mutations and result in secondary deregulation of the epigenome (Kobow and Blumcke, 2011). Gene promoters from mTLE patients are characterized by altered DNA methylation patterns and decreased DNA methyltransferase (Dnmt) gene expression (Kobow et al., 2009; Zhu et al., 2012).

**FIGURE 2:**
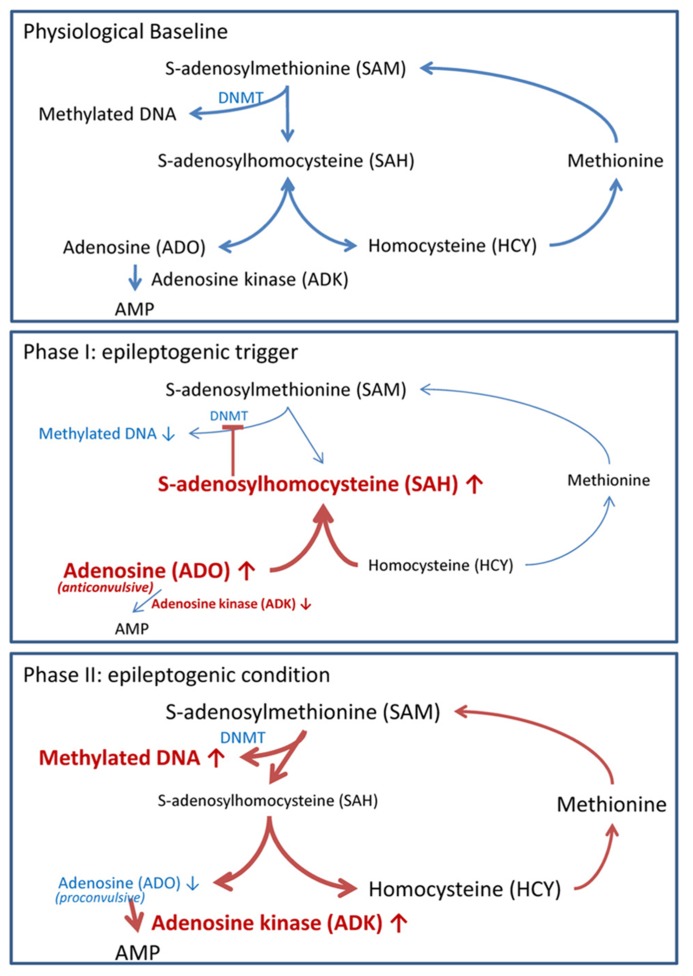
** Adenosine tone regulates the transmethylation pathway.** Pathways in blue reflect steady state pathways, whereas pathways in red show pathway shifts induced by alteration of adenosine homeostasis. Physiological baseline: DNA methylation of cytosine residues is mediated by the transmethylation pathway. *S*-adenosylmethionine (SAM) donates a methyl group, which is added to cytosine residues by DNMT. In the process SAM is converted to *S*-adenosylhomocysteine (SAH). SAH is further converted to adenosine (ADO) and homocysteine (HCY) by the enzyme *S*-adenosylhomocysteine hydrolase. ADO is phosphorylated to AMP by the enzyme adenosine kinase (ADK). HCY is converted to methionine and subsequently back to SAM. DNA methylation is dependent on the constant removal of the obligatory endproducts ADO and HCY. Phase I (epileptogenic trigger): we hypothesize that the injury and/or SE induced decrease of ADK and surge of ADK (Clark et al., 1997; Gouder et al., 2004; Pignataro et al., 2008) shifts the equilibrium constant of the transmethylation pathway to SAH. The increased SAH prevents SAM donation of a methyl group to DNA. Reduced DNA methylation permits the transcription of early epileptogenesis genes. Phase II (epileptogenic condition): increased ADK within reactive astrocytes reduces adenosine tone to pathologically low levels. Low adenosine tone shifts the biochemical pathway to favor SAM conversion to SAH; thereby, DNA methylation will be increased. Pathological hypermethylation of DNA is present in resected hippocampi of mTLE with hippocampal sclerosis patients (Kobow et al., 2009).

Biochemically, DNA methylation is intricately linked to *S*-adenosylmethionine (SAM)-dependent transmethylation reactions (**Figure 2**). SAM donates a methyl group to unmethylated cytosines in DNA. Following methyl group donation, SAM is converted to *S*-adenosylhomocysteine (SAH). SAH is further hydrolyzed to adenosine and homocysteine (HCY). Adenosine is cleared by ADK-mediated phosphorylation to AMP, and HCY is converted to methionine in a folate-dependent manner. Importantly, DNA methylation is dependent on the continuous removal and subsequent equilibrium constants of SAH, adenosine and HCY (Lu, 2000; Boison et al., 2002). Thus, an accumulation of adenosine prevents the biochemical conversion of SAM to SAH and therefore inhibits DNA methylation.

Humans with ADK deficiency caused by a missense mutation in the ADK gene have disruptions to the transmethylation pathway with increased methionine and SAH levels. Furthermore, they have abnormal liver function; encephalopathy; and severe progressive neurological deficits (Bjursell et al., 2011). Transgenic mice with an ADK knockout also have disruptions in the transmethylation pathway with decreased blood adenine levels and increased HCY levels and the fatal liver disease neonatal hepatic steatosis (Boison et al., 2002). Peripheral changes in the transmethylation pathway are conserved within the brain. We recently found that hippocampal adenosine levels regulate the global DNA methylation status by shifting the equilibrium constant of the transmethylation pathway; thereby either increasing (high ADK and low adenosine) or decreasing (low ADK and high adenosine) methylation. These adenosine dependent changes in DNA methylation are receptor-independent and can be evoked by either a single pharmacological bolus of adenosine (icv) or in response to endogenous changes in ADK expression or activity (Williams-Karnesky et al., 2013). As an obligatory endproduct of transmethylation, the adenosine tone non-specifically drives the transmethylation pathway by regulating substrate availability; with site specific DNA methylation mediated by DNMT1, 3a or 3b complexes (Goll and Bestor, 2005; Caiafa et al., 2009; Feng et al., 2010; Zampieri et al., 2012). Consequently, the adenosine tone does not regulate site specific DNA methylation, but instead the homeostasis of the DNA-methylome.

## THE ADENOSINE KINASE HYPOTHESIS OF EPILEPTOGENESIS

As outlined above, glial pathologies play important roles in epileptogenesis. Astrocytes form the major metabolic reuptake route for synaptic adenosine and control the availability of extracellular adenosine via expression changes of the astrocyte-based enzyme ADK, which phosphorylates adenosine to AMP and thereby drives the influx of adenosine into the astrocyte through equilibrative transporters (Boison, 2013). Using transgenic approaches and adenosine microelectrode biosensors we previously demonstrated that ADK expression levels in astrocytes directly control the levels of tissue adenosine under baseline conditions (Etherington et al., 2009). During epileptogenesis the adenosine/ADK system undergoes biphasic changes that might be instrumental in epileptogenesis and seizure generation (**Figure 2**).

### PHASE I OF ADENOSINE DYSREGULATION

Injuries to the brain such as trauma, stroke, or SE trigger an acute surge in adenosine, which is accompanied by transient downregulation of ADK (Clark et al., 1997; Pignataro et al., 2008). The initial injury and the associated surge in adenosine can trigger several mechanisms possibly implicated in epileptogenesis, among which the induction of A_2A_R expression in glial cells appears to play a prominent role. In primary cultures of glial cells lipopolysaccharide (LPS) was found to induce A_2A_R mRNA and protein expression with a peak at 48 h after treatment (Saura et al., 2005). Likewise, in microglial cells and astrocytes of the mouse substantia nigra 1-methyl-4-phenyl-1,2,3,6-tetrahydropyridine (MPTP) induced the expression of A_2A_Rs within 24 h after intoxication. The proliferation of astrocytes and thereby the development of astrogliosis is in part regulated by the ratio of the different adenosine receptors expressed on the astrocyte membrane. Importantly, the increased activation of A_2A_Rs by an injury-associated surge in adenosine can increase astrocyte proliferation and activation, whereas the blockade of A_2A_Rs prevented the induction of astrogliosis by BDNF, which is a known transactivator of the A_2A_R (Hindley et al., 1994; Brambilla et al., 2003; Rajagopal et al., 2004). In addition, important immune functions of the brain are under the control of adenosine homeostasis (Hasko et al., 2005, 2008). Via simultaneous activation of A_1_ and A_2A_ receptors adenosine was shown to stimulate the proliferation of naïve microglial cells (Gebicke-Haerter et al., 1996), whereas the A_2A_ receptor-dependent upregulation of cyclooxygenase 2 (COX-2) and the release of prostaglandin E2 (PGE2) were shown to mediate additional pro-inflammatory effects of adenosine (Fiebich et al., 1996). Multiple inflammatory processes, which have been linked to epileptogenesis (Ravizza et al., 2011) could be triggered by an injury-induced surge in adenosine. Recent findings also demonstrate that increased levels of adenosine induce hypomethylation of hippocampal DNA as described previously by shifting the equilibrium constant of the transmethylation pathway (Williams-Karnesky et al., 2013). The reduced methylation of CpG rich promoter regions could induce the transcription of epileptogenesis genes, suggesting a novel mechanism whereby an acute injury-induced surge in adenosine could trigger epileptogenesis.

### PHASE II OF ADENOSINE DYSREGULATION

Astrogliosis is a pathological hallmark of the human and experimental epileptic brain and has consistently been associated with overexpression of ADK resulting in adenosine deficiency (Li et al., 2008; Aronica et al., 2013). Adenosine deficiency in human epilepsy has directly been identified via the analysis of microdialysis samples. A 25% reduction of adenosine in the epileptogenic versus the contralateral control hippocampus was found (During and Spencer, 1992). In our prior work we provided the following evidence linking expression levels of ADK to seizure propensity: during epileptogenesis increased ADK expression and emergence of spontaneous electrographic seizures coincided both temporally as well as spatially (Li et al., 2008). The transgenic overexpression of ADK (40% increase) in the brain of mice triggered spontaneous electrographic seizures (Li et al., 2008), whereas a transgenic approach that reduced ADK expression in cortex and hippocampus of mice (40% reduction) rendered those animals resistant to seizures and resistant to epileptogenesis (Li et al., 2008). These data demonstrate that ADK provides a molecular link between astrogliosis and increased neuronal excitability.

In addition, the epileptogenic hippocampus is characterized by increased DNA methylation. This hypermethylated state forms the basis of the methylation hypothesis of epileptogenesis, which suggests that seizures by themselves can induce epigenetic chromatin modifications and thereby aggravate the epileptogenic condition (Kobow and Blumcke, 2011). Hypermethylation of DNA can be triggered by a variety of mechanisms, however, the mechanisms underlying the gradual increase in DNA methylation status during the course of epileptogenesis are not well characterized and largely the subject of speculation (Kobow and Blumcke, 2012). The epigenetic drift hypothesis suggests that a gradual shift in the ratio of active DNA demethylation and *de novo* methylation, triggered by a precipitating injury and modified by environmental and intrinsic factors leads to increased DNA methylation, altered gene expression, and an altered (e.g., seizure) phenotype (Feil and Fraga, 2011). We propose that overexpression of ADK in the epileptogenic hippocampus and resulting adenosine deficiency drives the biochemical transmethylation pathway and thereby increases the methylation rate of the hippocampal DNA. It is important to note that adenosine affects DNA methylation in a non-cell-autonomous manner and thereby is uniquely positioned to effect homeostasis of the DNA-methylome on a global scale within the hippocampal formation (Williams-Karnesky et al., 2013). Through this mechanism, astrogliosis and associated overexpression of ADK could contribute to continued epileptogenesis through maintenance of a hypermethylated state of hippocampal DNA. Conversely, reduction of DNA methylation through therapeutic adenosine augmentation may provide a rational therapeutic approach for the prevention of epileptogenesis.

## ANTIEPILEPTOGENIC THERAPIES

Several lines of evidence suggest that adenosine might prevent epileptogenesis. Transgenic mice with an engineered reduction of ADK expression in forebrain were found to be resistant to the development of epilepsy, even when the epileptogenesis-triggering SE was coupled with transient blockade of the A_1_R (Li et al., 2008). Similarly, adenosine-releasing stem cells – implanted into the hippocampal formation after triggering epileptogenesis – dose-dependently attenuated astrogliosis, suppressed ADK increases, and attenuated development of spontaneous seizures (Li et al., 2008). Using an independent therapeutic approach, the transient delivery of adenosine by intraventricular silk for only 10 days provided long-lasting (beyond adenosine release) antiepileptogenic effects in the rat kindling model of epilepsy (Szybala et al., 2009). More recent findings, as will be discussed in more detail below, suggest that the antiepileptogenic effects of adenosine are based on an epigenetic mechanism. Since dietary interventions have been shown to increase adenosine signaling in the brain (Masino et al., 2011), dietary manipulations such as the ketogenic diet might likewise hold promising therapeutic potential for the prevention of epileptogenesis.

### EPIGENETIC THERAPIES

As mentioned previously, DNA methylation has been highlighted as a component of the methylation hypothesis of epileptogenesis (Kobow and Blumcke, 2011). Consequently, DNA methylation inhibitors might be of therapeutic value to either treat epilepsy by restoring non-pathological epigenetic homeostasis. Unfortunately, the use of DNMT inhibitors for treating epileptic patients must be approached with caution due to target related complications or side effects. As an alternative to conventional pharmacological DNMT inhibitors focal adenosine therapy may serve as an effective epigenetic medicine. Recently, we described a novel antiepileptogenic role for adenosine; whereby a transient adenosine augmentation therapy administered to epileptic rats after the onset of spontaneous recurrent seizures not only suppressed seizures during active adenosine release, but also prevented further disease progression that lasted long after the therapy was suspended. Adenosine-dependent changes in DNA methylation were pinpointed as an underlying mechanism for the antiepileptogenic properties of this adenosine therapy. Adenosine treatment was found to restore normal DNA methylation levels in the otherwise hypermethylated hippocampus of the epileptic rat. More specifically, genome wide analysis using a methylated DNA immunoprecipitation (MeDIP) array revealed that out of the 125 genes which showed increased DNA methylation in epilepsy, 66 also showed reduced DNA methylation after adenosine therapy in treated epileptic rats. Interestingly, multiple targets that function to either interact with DNA or play a role in gene transcription and translation (*PolD1, Polr1e, Rps6kl1, Snrpn, Znf524, Znf541, Znf710*) responded to adenosine therapy with a decrease in the DNA methylation status of their respective promoters. Consequently, these targets are poised as likely candidates to mediate adenosine-dependent changes in major homeostatic functions (Williams-Karnesky et al., 2013).

### DIETARY INTERVENTIONS

Dietary therapies for epilepsy can be useful in cases where medications and other treatments are ineffective. The most obvious application is for epilepsies associated with specific inborn errors of metabolism that can be treated by removing or adding specific dietary components. Such disorders include dysfunctions of the enzymes phenylalanine hydroxylase, argininosuccinate synthetase, guanidinoacetate methyltransferase, 3-methylcrotonyl-CoA carboxylase, antiquitin, or various enzymes and transporters for metabolizing different classes of lipids (Papetti et al., 2012). Such disorders range from somewhat to extremely rare.

For more common forms of epilepsy, one effective dietary treatment is also the simplest: no diet at all. Fasting has been known to alleviate convulsions since antiquity. Some of the first work in the modern era on this topic was done by Geyelin in the 1910s (Geyelin, 1921). In working with grand mal and petit mal patients of various ages, he found that 22 out of 26 subjects were seizure-free after 10 days of fasting, with many experiencing beneficial effects after just 2 days of fasting. Furthermore, two subjects remained seizure-free for a year after the end of fasting, suggesting the potential for epigenetic and antiepileptogenic effects. Yet fasting has a number of problems, including the difficulty of maintaining a fast and being necessarily time-limited. Also, fasting would be contraindicated in patients that have other health problems besides epilepsy, growing children, and adults with very low body mass index.

Fasting forces a metabolic shift in which the liver metabolizes fatty acids into ketone bodies, which in the absence of sufficient glucose can be used as fuel by other tissues, particularly the brain, which is glucose-dependent. Hypothesizing that the antiseizure effects of fasting were due to this state of ketosis, in the 1920s Wilder began to use in his epileptic patients a strict modified diet which also produces ketosis (Wilder, 1921; Wilder and Winter, 1922). This ketogenic diet, low in carbohydrate, high in fat, with moderate-to-low protein, thus reproduces a major metabolic effect of fasting while still allowing food and was found to be an effective anticonvulsant in children and adults (McQuarrie and Keith, 1927; Baborka, 1930). Similar to fasting, initial reports also suggested an antiepileptogenic potential of a dietary approach, but there has been little research to follow up on using controlled studies.

The ketogenic diet was a fairly common epileptic treatment until the synthesis of phenytoin and other effective anticonvulsant drugs in the 1930s, which were easier to implement than a strict diet. Notably, anticonvulsant drugs often have sedative and cognitive side effects that the ketogenic diet lacks (Gupta et al., 2001; Drane and Meador, 2002). Thus, the ketogenic diet became a rare treatment, until a slow resurgence began in the 1990s in its use mostly for drug-refractory pediatric epilepsy. Often the diet now is supplemented with medium-chain triglycerides, which are absorbed quickly and metabolized easily to ketones. Good success rates were reported in retrospective (Kinsman et al., 1992; Hassan et al., 1999) and prospective studies (Freeman et al., 1998; Vining et al., 1998).

The first randomized, controlled study of the ketogenic diet clearly demonstrated a beneficial effect (Neal et al., 2008). These studies often showed efficacy equal to anticonvulsant drugs and while seizures worsened as a whole in patients on anticonvulsant medications they improved as a whole in the group on the diet. While a subset of the children on the ketogenic diet became seizure-free, this did not occur in the group receiving standard therapy with anticonvulsants. Even though this study was randomized and controlled, blinded studies of the ketogenic diet present obvious difficulties; one attempt, with mixed results, has been published (Freeman et al., 2009).

Multiple lines of evidence suggest that a ketogenic diet can increase ATP or mitochondrial biogenesis (Bough et al., 2006), and that it may exert anticonvulsant effects via adenosine (Masino et al., 2011). Further validation of these findings could identify the metabolic shift, which accompanies a ketogenic diet (or fasting), as a key strategy to increase adenosine and restore homeostasis. Along those lines, and as noted above, the potential for the ketogenic diet to restore homeostasis and offer antiepileptogenic activity deserves additional research, particularly as this dietary therapy is pursued for diverse neurological indications in which epilepsy is often comorbid.

## CONCLUSIONS AND OUTLOOK

Homeostasis has long been recognized as a core physiological principle, and the CNS depends critically on maintaining its milieu, including ion gradients, temperature, pH, and cell energy, as well as also regulating transcription and translation to ensure proper function. Chronic disruption of either its environment (the milieu) or its adaptive response to its environment (transcription and translation) results in a loss of CNS homeostasis and pathological dysfunction. It becomes clear that complex neurological syndromes, such as epilepsy, which are not only defined by a dominant symptom (i.e., a seizure), but also by a growing number of associated comorbidities, can best be explained by the disruption of network homeostasis. Disruption of network homeostasis will lead to the dysregulation of several molecular pathways (e.g., those dependent on K^+^, glutamate, and adenosine homeostasis) simultaneously. It becomes clear that conventional drugs with a mode of action that is restricted to only one target or pathway might be sufficient to block a symptom (e.g., a seizure), but are unlikely to affect a neurological condition on the network level. Novel therapeutic interventions based on adenosine, epigenetic mechanisms, or dietary interventions might hold promise to affect network homeostasis as a novel conceptual strategy to treat and prevent epilepsy on the network level. For future therapy development it is important to note that adenosine augmentation has no known adverse effects. In preclinical toxicity studies of intrathecal adenosine in dogs, no side effects were observed with intrathecal adenosine infused chronically for 26 days (Chiari et al., 1999). Likewise, intrathecal adenosine was tested in humans in escalating doses of up to 2 mg without any adverse effects (Eisenach et al., 2002a,b). Importantly, suprahippocampal implants of adenosine-releasing cells demonstrated a robust pro-cognitive effect in mice (Shen et al., 2012), suggesting that therapeutic adenosine augmentation might combine anticonvulsant with cognition-enhancing effects. Whereas seizure suppression in chronic epilepsy would require continuous long-term augmentation of adenosine, e.g., by gene therapy, cell grafts, or dietary intervention, preventing disease progression in the early stages of epilepsy might require only the transient delivery of adenosine. Adenosine-releasing silk might be an attractive therapeutic candidate due to the bioresorbable properties of this biopolymer. Those and related approaches are currently in preclinical development and molecular pathways stimulated by adenosine augmentation are currently under intense investigation.

## Conflict of Interest Statement

The authors declare that the research was conducted in the absence of any commercial or financial relationships that could be construed as a potential conflict of interest.
